# Guanabenz Downregulates Inflammatory Responses via eIF2α Dependent and Independent Signaling

**DOI:** 10.3390/ijms17050674

**Published:** 2016-05-05

**Authors:** Shinya Takigawa, Andy Chen, Akinobu Nishimura, Shengzhi Liu, Bai-Yan Li, Akihiro Sudo, Hiroki Yokota, Kazunori Hamamura

**Affiliations:** 1 Department of Biomedical Engineering, Indiana University Purdue University Indianapolis, Indianapolis, IN 46202, USA; wonderfulmexicancombo@hotmail.com (S.T.); meiten0903@gmail.com (A.N.); liu441@iupui.edu (S.L.); hyokota@iupui.edu (H.Y.); 2 Department of Orthopadic Surgery, Mie University Graduate School of Medicine, Mie 514-8507, Japan; a-sudou@clin.medic.mie-u.ac.jp; 3 Weldon School of Biomedical Engineering, Purdue University, West Lafayette, IN 40907, USA; andychen@iupui.edu; 4 Department of Pharmacology, School of Pharmacy, Harbin Medical University, Harbin 150081, China; liby@ems.hrbmu.edu.cn; 5 Department of Pharmacology, School of Dentistry, Aichi-Gakuin University, 1–100 Kusumoto-cho, Chikusa-ku, Nagoya 464-8650, Japan

**Keywords:** guanabenz, microarray, inflammation, Csf2 (GM-CSF), eIF2α signaling

## Abstract

Integrated stress responses (ISR) may lead to cell death and tissue degeneration via eukaryotic translation initiation factor 2 α (eIF2α)-mediated signaling. Alleviating ISR by modulating eIF2α phosphorylation can reduce the symptoms associated with various diseases. Guanabenz is known to elevate the phosphorylation level of eIF2α and reduce pro-inflammatory responses. However, the mechanism of its action is not well understood. In this study, we investigated the signaling pathway through which guanabenz induces anti-inflammatory effects in immune cells, in particular macrophages. Genome-wide mRNA profiling followed by principal component analysis predicted that colony stimulating factor 2 (Csf2, or GM-CSF as granulocyte macrophage colony stimulating factor) is involved in the responses to guanabenz. A partial silencing of Csf2 or eIF2α by RNA interference revealed that Interleukin-6 (IL6), Csf2, and Cyclooxygenase-2 (Cox2) are downregulated by guanabenz-driven phosphorylation of eIF2α. Although expression of IL1β and Tumor Necrosis Factor-α (TNFα) was suppressed by guanabenz, their downregulation was not directly mediated by eIF2α signaling. Collectively, the result herein indicates that anti-inflammatory effects by guanabenz are mediated by not only eIF2α-dependent but also eIF2α-independent signaling.

## 1. Introduction

Varying cellular stresses such as oxidation, nutrient deprivation, and stress to the endoplasmic reticulum induce integrated stress responses (ISR), in which cells attempt to withstand adverse environments in part by reducing the rate of protein synthesis [1]. Phosphorylated eukaryotic translation initiation factor 2 α (eIF2α) contributes to lowering protein production, and chemical compounds that block de-phosphorylation of eIF2α are known to suppress the cell death associated with ISR [2]. Two such agents, salubrinal (479.8 Da) and guanabenz (231.1 Da), bind to a subunit of protein phosphate 1 (PP1) complex and elevate the level of eIF2α phosphorylation [3,4]. We have previously shown that both salubrinal and guanabenz activate bone-forming osteoblasts via eIF2α-mediated upregulation of activating transcription factor 4 (ATF4), one of the three known transcription factors for bone formation [5]. Furthermore, they inhibit development of bone-resorbing osteoclasts by inactivating nuclear factor of activated T-cells cytoplasmic 1 (NFATc1), a master transcription factor of osteoclastogenesis [5,6,7].

In spite of the functional commonality of salubrinal and guanabenz in skeletal tissues, their chemical structures are dissimilar and their effects on inflammatory responses are largely different [8]. For instance, salubrinal downregulates the expression and activity of matrix metalloproteinase 13 (MMP13) in chondrocytes, and this downregulation is mediated via p38 and NFκB signaling [9]. Interestingly, salubrinal-driven regulation of p38 and NFκB is not linked to the phosphorylation level of eIF2α, and guanabenz does not present an inhibitory effect to MMP13 [10]. Besides acting as an inhibitor of PP1 complex, guanabenz serves as an agonist of α2 adrenergic receptor, and it has been FDA-approved for treatment of hypertension [11]. However, salubrinal does not have any known link to adrenergic signaling.

Recently guanabenz has been reported to have a potential therapeutic effect for multiple sclerosis, which is an inflammatory disease associated with oligodendrocyte death in the central nervous system [12]. It is proposed that enhancement of ISR activity by guanabenz contributes to damping inflammatory responses and protecting cytokine-mediated cell death [12,13]. The study herein aimed to understand the regulatory mechanism of anti-inflammatory actions by guanabenz in the immune cells, in particular macrophages, paying attention to eIF2α-dependent and eIF2α-independent signaling pathways.

In this study, we conducted *in vitro* analysis using four sources of immune cells (RAW264.7 macrophages, primary macrophages, Jurkat T lymphocytes, and HMC-1.1 mast cells). The effects of guanabenz on inflammatory responses were evaluated through genome-wide microarray experiments followed by a principal component analysis (PCA) [14]. PCA highlighted a set of genes that were most significantly affected by administration of guanabenz, including early growth response protein 2 (Egr2) [15] and colony-stimulating factor 2 (Csf2; also known as granulocyte macrophage colony-stimulating factor, GM-CSF) [16]. We employed RNA interference as well as salubrinal (inhibitor of de-phosphorylation of eIF2α), and searched potential regulatory pathways involved in guanabenz-driven anti-inflammatory responses.

## 2. Results

### 2.1. Guanabenz-Driven Suppression of Inflammatory Gene Expression

**Figure 1:**
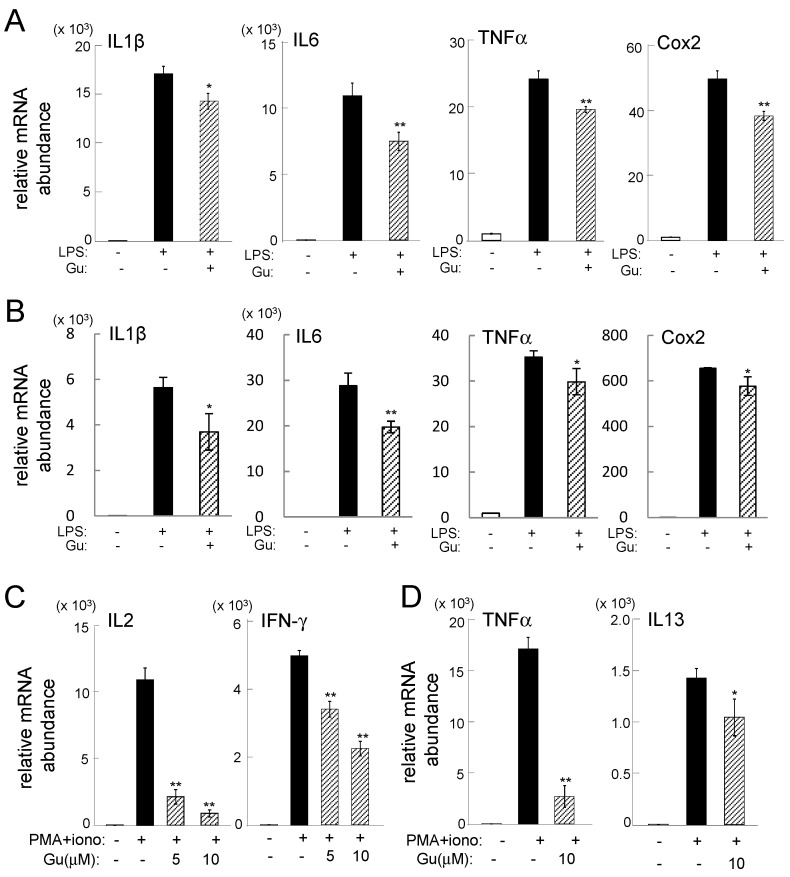
Guanabenz-driven suppression of inflammatory gene expression. The single and double asterisks indicate *p* < 0.05 and *p* < 0.01, respectively. (**A**) Levels of IL1β, IL6, TNFα, and Cox2 mRNAs in LPS-stimulated RAW264.7 cells in response to 10 µM guanabenz; (**B**) levels of IL1β, IL6, TNFα, and Cox2 mRNAs in LPS-stimulated primary macrophages in response to 10 µM guanabenz; (**C**) levels of IL2 and IFNγ mRNAs in PMA-stimulated Jurkat cells in response to 5 and 10 µM guanabenz; (**D**) levels of TNFα and IL13 mRNAs in PMA-stimulated HMC1.1 cells in response to 10 µM guanabenz.

Lipopolysaccharide (LPS)-stimulated RAW264.7 cells and mouse primary macrophages elevated the mRNA levels of IL1β, IL6, TNFα, and Cox2, but their elevation was significantly reduced by administration of 10 µM guanabenz for 6 h (Figure 1A,B). The mRNA levels of IL2 and IFNγ were increased in phorbol myristate acetate (PMA)-stimulated Jurkat cells, while those of TNFα and IL13 were upregulated in PMA-stimulated HMC1.1 cells. However, administration of 5 or 10 µM guanabenz significantly suppressed the upregulated mRNA levels (Figure 1C,D).

### 2.2. Prediction of the Pathways and Genes Involved in the Responses to Guanabenz

Genome-wide mRNA expression analysis predicted signaling pathways that were potentially involved in the responses to guanabenz for 6 h in LPS-stimulated macrophages (Table 1). Those pathways included inflammation-related pathways such as cytokine and chemokine signaling, and rheumatoid arthritis, as well as macrophage-linked pathways such as T-cell receptor signaling and hematopoietic cell lineage.

**Figure 2:**
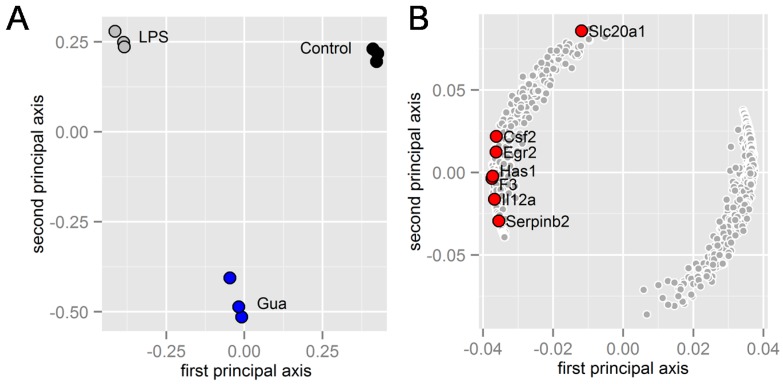
Microarray-based prediction of regulatory genes in response to guanabenz. (**A**) separation of three groups (control, LPS, and LPS + guanabenz) on the first and second principal plane (sample plane); (**B**) locations of the genes significantly altered by LPS and guanabenz on the first and second principal plane (gene plane). The genes marked in red were upregulated by LPS, and significantly downregulated by guanabenz.

In the primary and secondary principal axes in the sample plane, the sample groups (control, LPS, LPS and guanabenz) were separated as three distinctive clusters, in which the LPS + guanabenz group (Gua) partially restored the negative effect of the LPS group along the first principal axis (Figure 2A). In the corresponding gene plane, the genes with the most negative values in the first principal axis are highlighted (Figure 2B). Among these genes, we focused on *Egr2* and *Csf2* (GM-CSF) since their LPS-driven upregulation was significantly downregulated by guanabenz (Table 2).

### 2.3. Guanabenz-Driven Downregulation of Egr2 and Csf2 in Lipopolysaccharide (LPS)-Stimulated Cells

**Figure 3:**
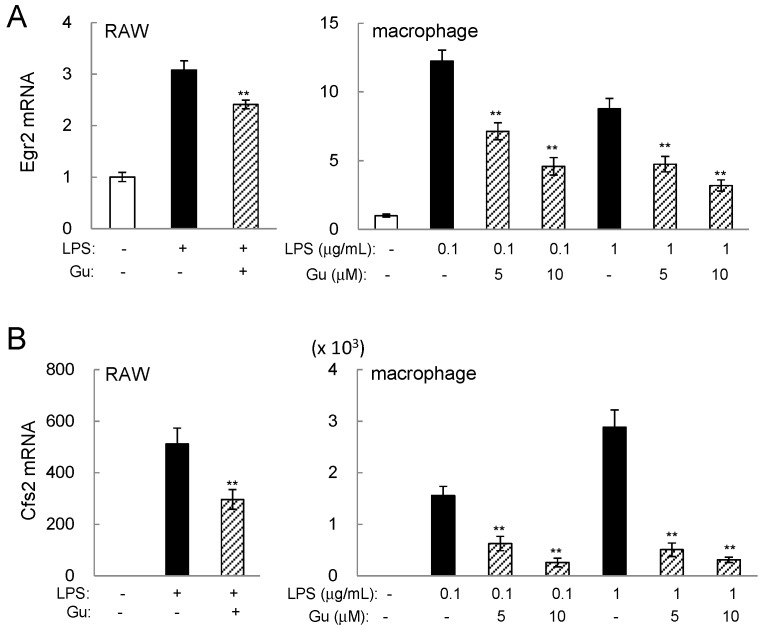
Guanabenz-driven downregulation of Egr2 and Csf2 (GM-CSF) in LPS-stimulated cells. The double asterisks indicate *p* < 0.01. (**A**) Egr2 mRNA levels in LPS stimulated RAW264.7 cells and primary macrophages in response to 5 or 10 µM guanabenz; (**B**) Cfs2 mRNA levels in LPS stimulated RAW264.7 cells and primary macrophages in response to 5 or 10 µM guanabenz.

PCA prediction indicated potential involvement of Egr2 and Csf2 in guanabenz-driven suppression of pro-inflammatory genes. Examination of their mRNA levels in the presence and absence of 5 or 10 µM guanabenz for 6 h revealed that guanabenz significantly reduced the mRNA levels of Egr2 and Csf2 in LPS-stimulated RAW264.7 cells (Figure 3A,B). In response to 0.1 µg/mL LPS and 10 µM guanabenz, guanabenz suppressed Csf2 mRNA levels to 59% (RAW cells) and 18% (primary macrophages), and Egr2 mRNA level to 77% (RAW cells) and 37% (primary macrophages).

**Figure 4:**
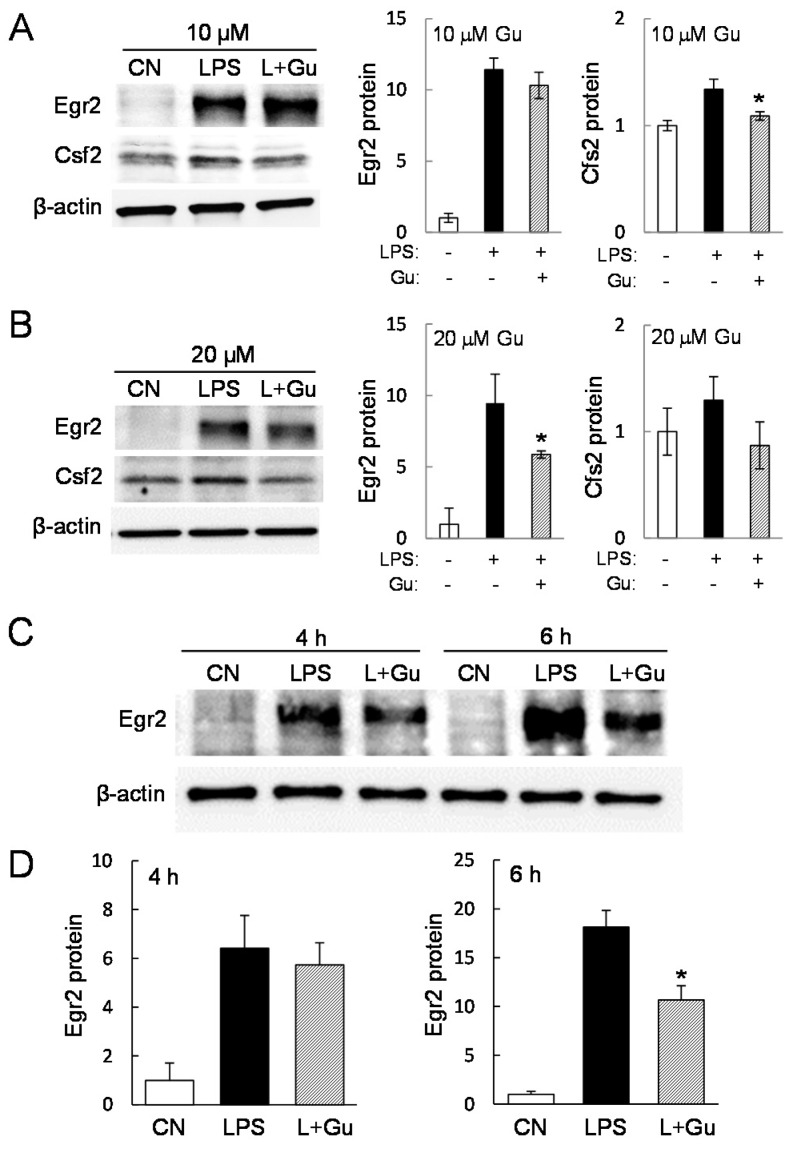
Alterations in the levels of Egr2 protein and Csf2 protein in response to guanabenz. The single asterisk indicates *p* < 0.05. (**A**,**B**) Egr2 and Csf2 protein levels in LPS-stimulated RAW264.7 cells in response to 10 and 20 µM guanabenz for 6 h, respectively; (**C**,**D**) Egr2 and Csf2 protein levels in LPS-stimulated primary macrophages in response to 10 µM guanabenz for 4 and 6 h.

In RAW264.7 cells, the LPS-induced level of Csf2 protein was significantly reduced by 6 h incubation with 10 µM guanabenz (Figure 4A). The LPS-induced protein level of Egr2 was significantly reduced not by 10 but by 20 µM guanabenz (Figure 4B). In primary macrophages, however, 10 µM guanabenz was sufficient to suppress LPS-induced elevation of Egr2 protein at 6 h (Figure 4C,D).

### 2.4. Potential Linkage of Egr2 and Csf2 to IL1β, TNFα, and Cox2

**Figure 5:**
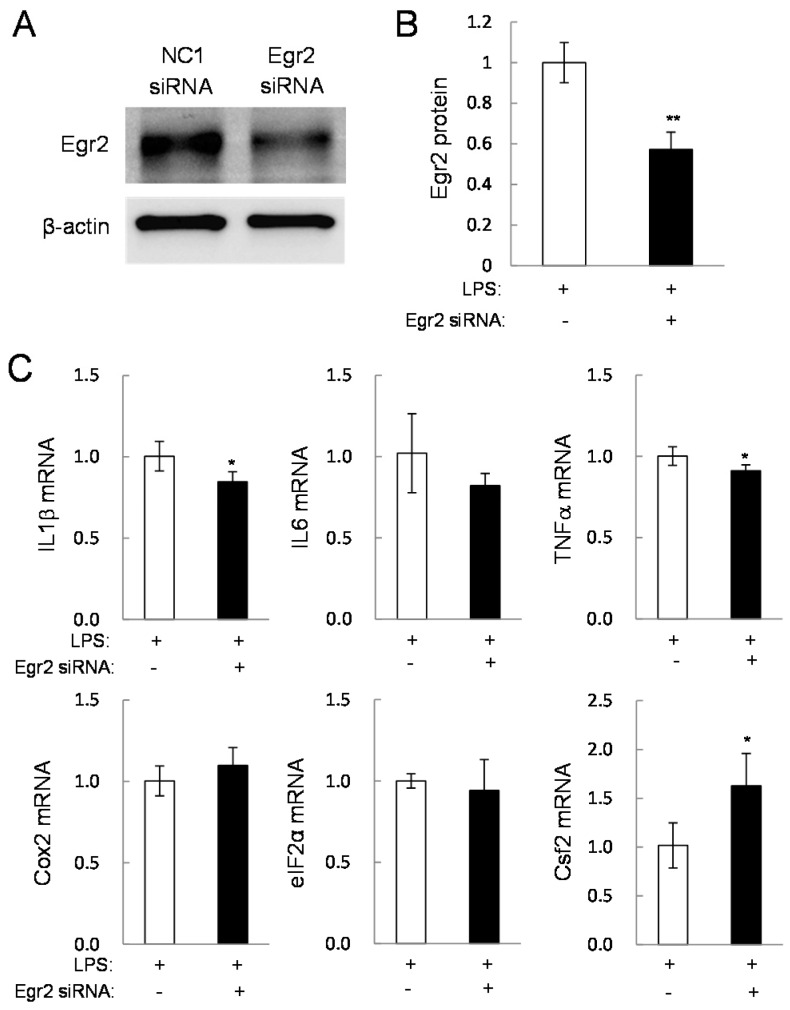
Effects of a partial silencing of Egr2 on the mRNA levels of the selected genes in LPS-stimulated RAW264.7 cells. The single and double asterisk indicates *p* < 0.05 and *p* < 0.01, respectively. (**A**,**B**) Reduction of Egr2 by treatment with Egr2 siRNA; (**C**) levels of IL1β, IL6, TNFα, Cox2, eIF2α, and Csf2 mRNAs in response to Egr2 siRNA treatment in LPS-stimulated RAW264.7 cells.

**Figure 6:**
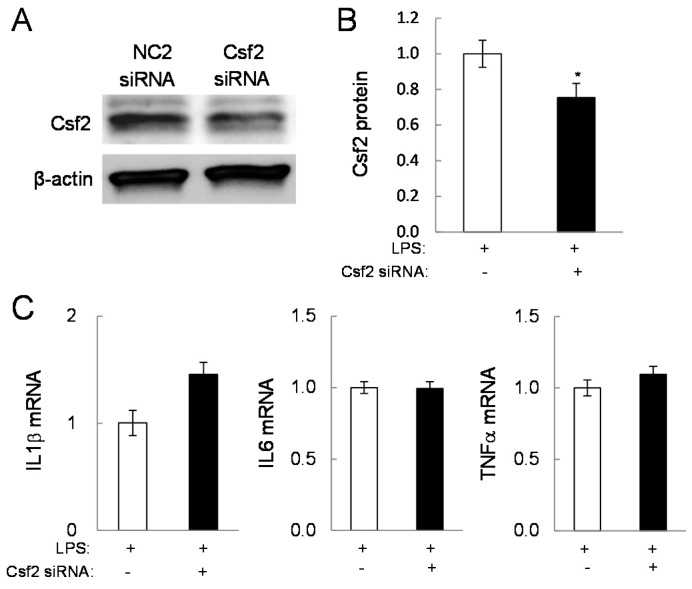

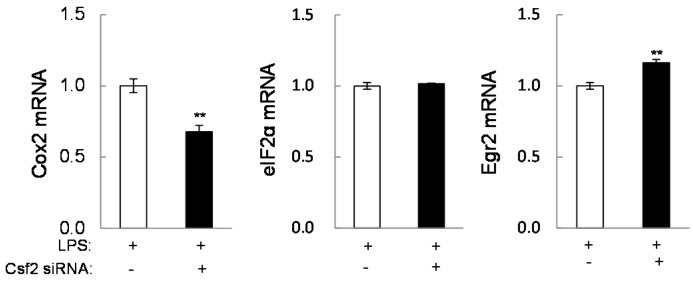
Effects of a partial silencing of Csf2 on the mRNA levels of the selected genes in LPS-stimulated RAW264.7 cells. The single and double asterisk indicates *p* < 0.05 and *p* < 0.01, respectively. (**A**,**B**) Reduction of Csf2 by treatment with Csf2 siRNA; (**C**) levels of IL1β, IL6, TNFα, Cox2, eIF2α, and Egr2 mRNAs in response to Csf2 siRNA treatment in LPS-stimulated RAW264.7 cells.

To examine the role of Egr2 and Csf2 in pro-inflammatory gene expression, their partial silencing was conducted using siRNAs in RAW264.7 cells. In response to Egr2 siRNA (Figure 5A,B), the mRNA levels of IL1β and TNFα were reduced and the mRNA level of Csf2 was elevated (Figure 5C). In response to Csf2 siRNA (Figure 6A,B), the Cox2 mRNA level was downregulated and the level of Egr2 mRNA was upregulated (Figure 6C). Neither siRNA altered the mRNA level of eIF2α.

### 2.5. Direct Linkage of eif2α Signaling to IL6, Cox2, and Csf2

**Figure 7:**
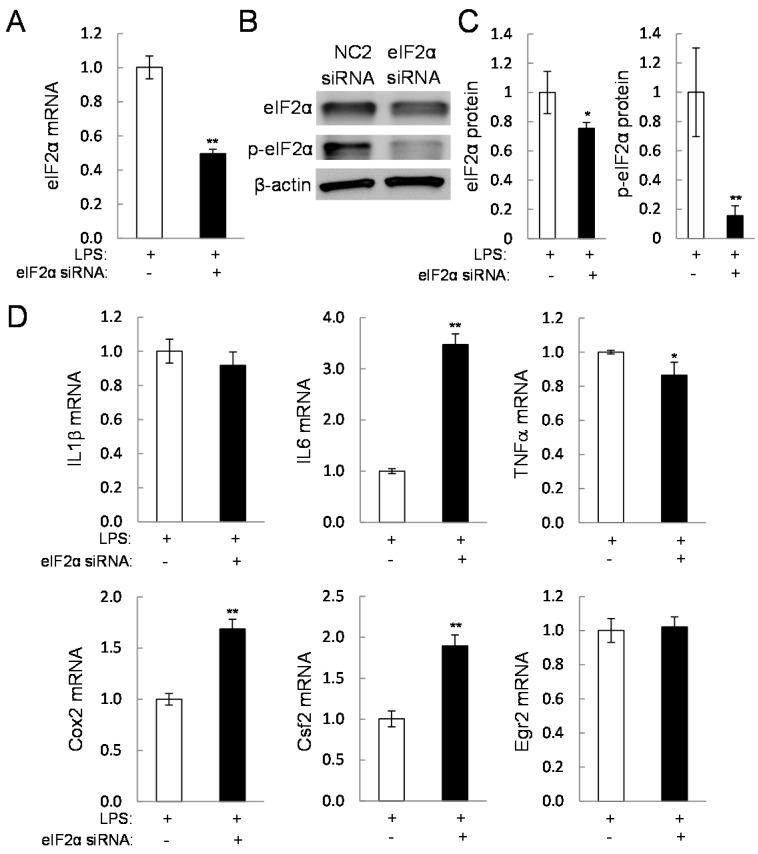
Effects of a partial silencing of eIF2α on the mRNA levels of the selected genes in LPS-stimulated RAW264.7 cells. The single and double asterisks indicate *p* < 0.05 and *p* < 0.01, respectively. (**A**–**C**) Reduction of eIF2 α and p-eIF2 α by treatment with eIF2 α siRNA; (**D**) levels of IL1β, IL6, TNF α, Cox2, Csf2, and Egr2 mRNAs in response to eIF2 α siRNA treatment in LPS-stimulated RAW264.7 cells.

**Figure 8:**
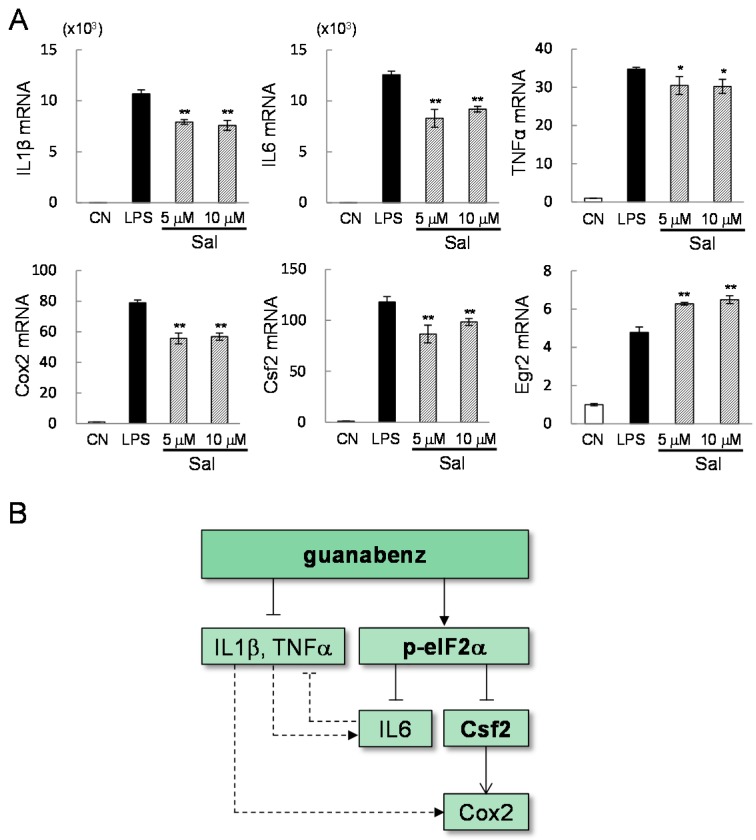
(**A**) Effects of salubrinal on the mRNA levels of the selected genes (IL1β, IL6, TNFα, Cox2, Csf2, and Egr2) in LPS-stimulated RAW264.7 cells. The single and double asterisks indicate *p* < 0.05 and *p* < 0.01, respectively; (**B**) Schematic mechanism of guanabenz-driven regulation of inflammatory genes. The solid line is based on the result in this study, while the dotted line is from other studies.

To evaluate the role of eIF2α signaling in the response to guanabenz, the protein level of eIF2α was reduced by RNA interference (Figure 7A–C). This partial silencing of eIF2α decreased not only the level of eIF2α but the level of phosphorylated eIF2α. The PCR results revealed that the mRNA levels of IL6 and Cox2, as well as Csf2, were significantly elevated by eIF2α siRNA (Figure 7D). However, the level of Egr2 mRNA was unchanged and a slight reduction in TNFα mRNA level was detected. In response to 5 or 10 µM salubrinal, LPS-driven upregulation of IL1β, IL6, TNFα, Cox2, and Csf2 mRNAs was significantly reduced (Figure 8A). Unlike the response to guanabenz, however, the level of Egr2 mRNA was further elevated by administration of 5 or 10 µM salubrinal.

## 3. Discussion

We present in this study that guanabenz is capable of downregulating the expression of IL1β, IL6, TNFα, and Cox2 in RAW264.7 and primary macrophages, IL2 and IFNγ in Jurkat lymphocytes, and TNFα and IL13 in HMC1.1 mast cells. Of note, the genes involved in inflammatory responses differ depending on the types of immune cells and our focus herein is macrophages. Genome-wide mRNA profiling in primary macrophages, followed by PCA and siRNA-based analyses in RAW264.7 cells, reveals that guanabenz-driven downregulation of IL6 and Cox2 is mediated by Csf2 in eIF2α-dependent signaling, while other inflammatory genes such as IL1β and TNFα are regulated independently from RNA interference with eIF2α siRNA. The levels of mRNA and protein were evaluated in response to 6 h incubation with guanabenz, unless otherwise specified.

Four genes such as IL1β, IL6, TNFα, and Cox2, which are responsive to guanabenz and primarily analyzed in this study, are coordinately expressed in a variety of immune responses. The schematic signaling pathway is depicted, in which the solid and dotted lines are based on this study and others [17,18,19], respectively (Figure 8B). It is reported, for instance, that IL1β activates Cox2, which converts arachidonic acid into prostaglandins and triggers the production of pro-inflammatory cytokines and chemokines [17]. IL6 is involved in the proliferation and differentiation of T-cells and B-cells in response to IL1 and TNF, but it also acts as anti-inflammatory cytokine and inhibits the functions of IL1 and TNF [18]. TNFα activates NFκB and MAPK signaling and promotes inflammatory responses, particularly against tumor cells [19].

The downregulation of Csf2, IL6, and Cox2 by guanabenz is mediated by eIF2α signaling. A partial silencing of eIF2α significantly elevates the expression levels of these three genes. Furthermore, RNA interference with Csf2 siRNA shows that downregulation of Csf2 is required for guanabenz-driven suppression of Cox2. Recently, Csf2 has been revealed as a potential therapeutic target in rheumatoid arthritis as well as multiple sclerosis, an inflammatory demyelinating disease in the central nervous system. A high level of Csf2 is detected in joints with rheumatoid arthritis [13], while an increase in Csf2-expressing B cells is detected in patients with multiple sclerosis [16]. Consistent with the results of this study, administration of guanabenz is reported to protect the nerve cells and alleviate clinical symptoms in a mouse model of multiple sclerosis [12].

While guanabenz and salubrinal are capable of suppressing LPS-driven upregulation of IL1β and TNFα, a partial silencing of eIF2α does not confirm that the effects of guanabenz are always mediated via eIF2α signaling. Guanabenz is known as an inhibitor of α2 adrenergic signaling [11], but treatment with clonidine, another α2 adrenergic agonist, did not alter expression of IL1β or TNFα (data not shown). We observed that Egr2 mRNA level was reduced by guanabenz and not by salubrinal. We also observed that a partial silencing of Egr2 by RNA interference significantly decreased the mRNA levels of IL1β and TNFα. Thus, it is possible that eIF2α-independent anti-inflammatory responses by guanabenz are mediated by Egr2. Egr2 is a transcription factor, and its mutation causes musculoskeletal diseases such as Charcot-Marie-Tooth disease and Dejerine–Sottas disease. It promotes the proliferation and survival of osteoprogenitor cells and maintains bone architecture [20]. However, its role in inflammation has not been well understood. In osteoblasts, expression of Egr2 is suppressed by glucocorticoids that are often administered for the management of autoimmune and inflammatory diseases [21]. Although both guanabenz and salubrinal elevate the level of eIF2α phosphorylation, they inhibit eIF2α’s phosphatase by binding to different subunits, and their potential link to Egr2 appears to differ.

Signaling pathways are dynamically regulated in time and space. In this study, we specifically analyzed the role of Csf2 and Egr2 from the list of seven genes in Table 2 since they are a cytokine and transcription factor, respectively. It is possible that other genes are also involved in the responses to guanabenz. To understand the complex signaling machinery, it is recommended to evaluate the interactive signaling in Figure 8 in a dynamical fashion using a range of guanabenz dosages. Although our microarray analysis and *in vitro* experiments were mostly conducted 6 h after incubation with guanabenz, gene expression at earlier or later time points may present other regulatory candidates. We have observed differential responses of Csf2 and Egr2 in RAW264.7 cells and primary macrophages. Further analysis is recommended to evaluate a potential cause of the observed variations, such as cellular differentiation stages and responsiveness to LPS, as well as the mechanism of the action of guanabenz in other immune cells.

## 4. Experimental Section

### 4.1. Cell Culture

RAW264.7 macrophages [22] and primary macrophages were cultured in αMEM with 10% FBS and antibiotics. Mouse bone marrow cells were grown with 10 ng/mL macrophage colony-stimulating factor (M-CSF, PeproTech, Rocky Hills, NC, USA) for three days, and the surface-attached cells were used as primary macrophages. Jurkat T lymphocytes [23] and HMC-1.1 mast cells [24] were cultured in RPMI 1640 and IMDM with 1-thioglycerol, respectively. RAW264.7 cells and primary macrophages were activated by 0.1 or 1 µg/mL lipopolysaccharide (LPS), while Jurkat cells and HMC-1.1 cells were activated by 100 nM phorbol myristate acetate (PMA) and 1 µM ionomycin. Of note, the MTT assay using RAW264.7 cells for evaluating cytotoxicity revealed that administration of guanabenz at 5, 10, and 20 µM for two days reduced relative cell growth down to 87%, 86%, and 75% of the control cells without guanabenz administration.

### 4.2. Microarray

Genome-wide expression analysis was conducted using RNA isolated from mouse primary macrophages (Mouse Gene 2.0 ST arrays, Affymetrix, Cleveland, OH, USA). The three groups (three samples per group) were “CN” (control), “LPS” (0.1 µg/mL LPS), and “Gua” (0.1 µg/mL LPS and 10 µM guanabenz). The samples were harvested 6 h after treatment with LPS and/or guanabenz. First, genes that were significantly modulated by LPS as well as guanabenz were identified. Then, signaling pathways that were over-represented in the selected genes were identified using WebGestalt software. Principal component analysis (PCA) was also performed, and genes that were likely to be involved in the responses to LPS and guanabenz were predicted. In brief, nine samples in three groups were positioned in the plane of the first and second principal axes, in which the first principal axis was identified as the axis for anti-inflammatory responses. Then, the genes whose mRNA levels were significantly altered along the first principal (anti-inflammatory) axis were selected for further *in vitro* evaluation.

### 4.3. qPCR and Western Blot Analysis

Reverse transcription was conducted using total RNA and a high-capacity cDNA reverse transcription kit (Applied Biosystems, Carlsbad, CA, USA). Quantitative real-time PCR was performed using Power SYBR green PCR master mix kits (Applied Biosystems). Using the PCR primers (Table 3), the mRNA levels of inflammatory genes (*IL1β*, *IL2*, *IL6*, *IL13*, *TNFα*, *IFNγ*, *Cox2*) as well as regulatory genes (*eIF2α*, *Csf2*, and *Egr2*) were determined with glyceraldehyde-3-phosphatedehydrogenase (GAPDH) as an internal control. Western blot analysis was conducted using 10%–12% sodium dodecyl sulfate (SDS) gels and polyvinylidene difluoride (PVDF) transfer membranes (Millipore, Billerica, MA, USA). Protein samples were isolated using a RIPA buffer. We used primary antibodies specific to Csf2 (Abcam, Cambridge, MA, USA), Egr2 (Proteintech, Rosemont, IL, USA), eIF2α and p-eIF2α (Cell Signaling, Danvers, MA, USA) as well as a secondary antibody conjugated with horseradish peroxidase (Cell Signaling). The level of proteins was detected using a SuperSignal west femto maximum sensitivity substrate (Thermo Scientific, Waltham, MA, USA), and the level of β-actin (Sigma, St. Louis, MO, USA) was employed as a control.

### 4.4. RNA Silencing Using siRNAs

RAW264.7 cells were transiently transfected with siRNA specific to Egr2, non-specific control siRNA #1 (Life Technologies, Grand Island, NY, USA), Csf2, eIF2α, or non-specific control siRNA #2 (GE Dharmacon, Lafayette, CO, USA) (Table 3), in Opti-MEM I medium using Lipofectamine RNAiMAX (Life Technologies). The efficiency of silencing was assessed by Western blotting 48 h after transfection.

### 4.5. Statistical Analysis

Three to four independent experiments were conducted, and data were expressed as mean ± S.D. Statistical significance was evaluated using Student’s *t*-test at *p* < 0.05; the single and double asterisks indicate *p* < 0.05 and *p* < 0.01, respectively.

## 5. Conclusions

This study demonstrates that administration of guanabenz reduces expression of Egr2 and Csf2 as well as inflammatory genes such as IL1β, IL6, TNFα, and Cox2 in macrophages in eIF2α-dependent and eIF2α-independent signaling.

## Figures and Tables

**Table 1:** Microarray-based prediction of pathways. Selected Kyoto Encyclopedia of Genes and Genomics (KEGG) pathways for primary macrophages.

Pathway	Ratio	*p*
Lysosome	3.27	0.0007
T cell receptor signaling pathway	3.29	0.0011
Hematopoietic cell lineage	3.70	0.0011
Cytokine-cytokine receptor interaction	2.38	0.0011
Fc gamma R-mediated phagocytosis	3.39	0.0017
Rheumatoid arthritis	3.39	0.0026
Chemokine signaling pathway	2.42	0.0044
MAPK signaling pathway	2.06	0.0077
ErbB signaling pathway	2.84	0.0154
NOD-like receptor signaling pathway	3.45	0.0154

**Table 2:** Heat map of the genes highlighted in Figure 2B.

Gene	Description	*p*1	C1	C2	C3	L1	L2	L3	G1	G2	G3
*F3*	coagulation factor III	−0.997									
*Has1*	hyaluronan synthase1	−0.992									
*Il12a*	interleukin 12a	−0.979									
***Egr2***	early growth response 2	−0.967									
***Csf2***	colony stimulating factor 2	−0.964									
*Serpinb2*	serine/cysteine peptidase inhibitor, B2	−0.946									
*Slc20a1*	solute carrier family 20, member 1	−0.317									

C1–C3, control samples; L1–L3, LPS samples; and G1–G3, Guanabenz-treated LPS samples. The blue and red colors indicate the downregulation and upreguation, respectively.

**Table 3:** Real-time polymerase chain reaction (PCR) primers and siRNAs used in this study.

Gene	Accession Number	Forward Primer	Backward Primer
**Mouse Primers**			
*IL1β*	NM_008361	5′-GCCCATCCTCTGTGACTCAT-3′	5′-AGGCCACAGGTATTTTGTCG-3′
*IL6*	NM_031168	5′-TTCCATCCAGTTGCCTTCTT-3′	5′-TCCACGATTTCCCAGAGAAC-3′
*TNFα*	NM_013693	5′-GAACTGGCAGAAGAGGCACT-3′	5′-AGGGTCTGGGCCATAGAACT-3′
*Cox2*	AF378830	5′-CCCCCACAGTCAAAGACACT-3′	5′-CTCATCACCCCACTCAGGAT-3′
*Csf2*	NM_009969	5′-GAGGCCATCAAAGAAGCCCT-3′	5′-AAATTGCCCCGTAGACCCTG-3′
*eIF2α*	NM_026114	5′-GAATGTACTCCAGATTGGCTGACTAC-3′	5′-CCTCAATGTGAAGACCTGTATCGA-3′
*Egr2*	NM_010118	5′-GATCTGCATGCGAAACTTCA-3′	5′-CACTGCTCTTCCGTTCCTTC-3′
*GAPDH*	NM_008084	5′-TGCACCACCAACTGCTTAG-3′	5′-GGATGCAGGGATGATGTTC-3′
**Human Primers**			
*IFNγ*	NM_000619	5′-TTCAGCTCTGCATCGTTTTG-3′	5′-TCTTTTGGATGCTCTGGTCA-3′
*IL2*	NM_000586	5′-GCAACTCCTGTCTTGCATTG-3′	5′-GCCTTCTTGGGCATGTAAAA-3′
*IL13*	NM_002188	5′-GTACTGTGCAGCCCTGGAAT-3′	5′-TTTACAAACTGGGCCACCTC-3′
*TNFα*	NM_000594	5′-CAGAGGGCCTGTACCTCATC-3′	5′-GGAAGACCCCTCCCAGATAG-3′
*GAPDH*	NM_001289745	5′-GCACCGTCAAGGCTGAGAAC-3′	5′-ATGGTGGTGAAGACGCCAGT-3′
**Mouse siRNAs**			
Csf2 (GM-CSF)		5′-GCGGAAGACAAACGAGAGA-3′	5′-GCCUGAAGAUAUUCGAGCA-3′
		5′-AUGAAGAGGUAGAAGUCGU-3′	5′-CCAGCUACUACCAGACAUA-3′
eIF2α (eIF2s1)		5′-UCGAGCAGAUAUUGAAGUA-3′	5′-CAUGAUUCUUCUUAGUGAA-3′
		5′-UGUCACAAGUUAAAGCCAA-3′	5′-GAACUCAAUGGGCAAGUAA-3′
Egr2		5′-CGACCUCGAAAGUACCCUA-3′	
Control #1 (NC1) for Egr2		5′-UGUACUGCUUACGAUUCGG-3′	
Control #2 (NC2) for Csf2 & eIF2α		5′-UGGUUUACAUGUCGACUAA-3′	5′-UGGUUUACAUGUUGUGUGA-3′
		5′-UGGUUUACAUGUUUUCUGA-3′	5′-UGGUUUACAUGUUUUCCUA-3′
